# Fcγ Receptor Type I (CD64)-Mediated Impairment of the Capacity of Dendritic Cells to Activate Specific CD8 T Cells by IgG-opsonized Friend Virus

**DOI:** 10.3390/v11020145

**Published:** 2019-02-08

**Authors:** Zoltán Bánki, Roland Werner, Lydia Riepler, Annika Rössler, Brigitte Müllauer, Verena Hegen, Wibke Bayer, J. Sjef Verbeek, Ulf Dittmer, Heribert Stoiber

**Affiliations:** 1 Division of Virology, Medical University of Innsbruck, Innsbruck, 6020, Austria; rol.w@gmx.net (R.W.); Lydia.Riepler@student.uibk.ac.at (L.R.); Annika.Roessler@i-med.ac.at (A.R.); brigitte.muellauer@i-med.ac.at (B.M.); verena.hegen@icloud.com (V.H.); heribert.stoiber@i-med.ac.at (H.S.); 2 Institute for Virology, University Hospital Essen, University of Duisburg-Essen, Essen, Germany; wibke.bayer@uni-due.de (W.B.); ulf.dittmer@uni-due.de (U.D.); 3 Department of Human Genetics, Leiden University Medical Center, Leiden, the Netherlands; J.S.Verbeek@lumc.nl (J.S.V.)

**Keywords:** friend virus, dendritic cells, IgG-opsonization, Fcγ receptors, CD8 T cells

## Abstract

Dendritic cells (DCs) express Fcγ receptors (FcγRs) for the binding immune complexes (ICs) consisting of IgG and antigens (Ags). IC–FcγR interactions have been demonstrated to enhance activation and antigen-presenting functions of DCs. Utilizing Friend virus (FV), an oncogenic mouse retrovirus, we investigated the effect of IgG-opsonization of retroviral particles on the infection of DCs and the subsequent presentation of viral antigens by DCs to virus-specific CD8 T cells. We found that opsonization by virus-specific non-neutralizing IgG abrogated DC infection and as a consequence significantly reduced the capacity of DCs to activate virus-specific CD8 T cells. Effects of IgG-opsonization were mediated by the high-affinity FcγR type I, CD64, expressed on DCs. Our results suggest that different opsonization patterns on the retroviral surface modulate infection and antigen-presenting functions of DCs, whereby, in contrast to complement, IgG reduces the capacity of DCs to activate cytotoxic T cell (CTL) responses.

## 1. Introduction

Fcγ receptors (FcγRs) provide a link between cellular and humoral immunity [1]. Depending on the presence of immunoreceptor tyrosine-based activation or inhibitory motif (ITAM or ITIM, respectively) in the intracellular region, FcγRs are divided into two groups: (i) activating FcγRI (CD64), FcγRIIa (CD32a), FcγRIII (CD16), and the recently identified FcγRIV or (ii) inhibitory FcγRIIb (CD32b) [2,3]. Of note, the genetic equivalent of the human activating FcγRIIa carrying an ITAM has not been found in the mouse [4]. The low-affinity FcγRII and FcγRIII mediate the binding of immune-complexed (IC) antigens (Ags), whereas FcγRI represents a high-affinity receptor for binding ICs and also monomeric IgG molecules [5].

Dendritic cells (DCs) are the most potent antigen-presenting cells (APCs). They capture and process antigens, which are presented to T cells. Due to their strong co-stimulatory capacity, mature DCs efficiently prime T cells recognizing presented Ags. DCs, both human and mouse, express several FcγRs including FcγRI, FcγRII, and at least in mouse FcγRIII [5,6]. The interaction of DCs with IgG immune-complexed with Ags has been demonstrated to modulate DC functions through these FcγRs. The engagement of FcγRs on DCs has been shown to induce maturation of DCs in both human and mouse through ITAM-associated activating FcγRs [7,8]. In contrast, FcγRIIb was observed to inhibit this ITAM-induced maturation [9]. ICs are internalized more efficiently by DCs compared to soluble Ags [10]. This FcγR-mediated uptake of Ags results in an improved Ag processing and presentation by DCs. Immune complex-derived Ags are presented by DCs and induce proliferation of Ag-specific major histocompatibility complex (MHC) class II-restricted CD4 T cells [11]. Furthermore, DCs are able to present exogenous Ags in an MHC class I context to CD8 T cells in a process referred to as cross-presentation. Such specific CD8 T cell responses are improved by immune-complexed Ags [6,12]. This enhancement of T cell responses by ICs relies on the presence of ITAM-bearing activating FcγRs [3,12,13]. In contrast to activating FcγRs, the role of inhibitory FcγRIIb in T cell responses is still controversial. In γ-chain knockout (KO) mice, solely expressing inhibitory FcγRIIb, conflicting results have been generated concerning the involvement of FcγRIIb in the IC-mediated presentation of Ags to T cells [12,13,14].

The immune-stimulatory role of FcγRs on DC-mediated CD8 T cell priming was mainly studied on immune-complexed protein Ags or tumor cells, and only a few studies focus on viral particles. We and others have shown that IgG-opsonization diminished HIV infection of human monocyte-derived DCs and attenuated complement-mediated enhancement of the capacity of DCs to activate virus-specific CD8 T cells [15,16]. However, the effect of IgG-opsonization and the role of FcγRs in the infection of APCs and subsequent Ag presentation are still not fully understood. To further investigate this aspect, we utilized a mouse retrovirus, Friend virus (FV), a well-established model to study retroviral infections. FV is a complex of two viruses: the non-pathogenic, helper Friend murine leukemia virus (F-MuLV) and the pathogenic, replication-deficient so-called spleen focus-forming virus (SFFV) [17]. Co-infection of adult mice with these two viruses leads to splenomegaly due to a polyclonal proliferation of erythroid precursor cells induced by the binding of the truncated envelope protein of SFFV to erythropoietin receptor (EpoR) on erythroid cells. In susceptible mouse strains, disease develops into lethal erythroleukemia. Disease-resistant strains can control acute infection, but the induction of regulatory T cells [18], myeloid-derived suppressor cells [19], as well as the expression of inhibitory receptors [20], lead to impaired functional activity of virus-specific cytotoxic T cells (CTLs) and a chronic infection develops [21,22].

Using this retroviral model, we show that FcγRI inhibits infection of mouse bone marrow-derived DCs (bmDC) by immune-complexed F-MuLV and subsequently abrogates presentation of viral antigens for FV-specific CD8 T cells in vitro.

## 2. Materials and Methods

### 2.1. Mice and Ethics Statement

Bone marrow-derived DCs were generated from 2- to 6-month-old C57BL/6 (Janvier Labs, Le Genest Saint Isle, France) mice. Breeding pairs of FcγRI^−/−^ [23], FcγRII^−/−^ [24], and FcγRIII^−/−^ [25] mice were obtained from the Department of Human Genetics, University of Leiden, Leiden, the Netherlands. Splenocytes were isolated from 2- to 6-month-old FV-specific CD8 T cell receptor (TCR) transgenic (tg) mice recognizing the gag leader-derived epitope GagL85-93 of FV [26] and ovalbumin (OVA)-specific CD8 TCRtg OT-1 (Janvier Labs) mice. All mice were bred and maintained free of specific pathogens in the animal facilities of the Medical University of Innsbruck. Mice were maintained according to the guidelines of the “European Convention for the Protection of Vertebrate Animals used for Experimental or other Scientific Purposes” and the Austrian Law.

### 2.2. Virus Stocks, Opsonization, and Virus Capture Assay (VCA)

F-MuLV stocks were generated in permissive *Mus dunni* cells. Virus-containing supernatants were harvested and stored at −80 °C until use. Focus-forming units (FFUs) of F-MuLV stocks were determined using *Mus dunni* cells in an infectious center assay (ICA). Alternatively, real-time quantitative RT-PCR with FV-specific forward- and reverse-primers as well as a fluorescent-labelled probe were performed to quantify DNA transcribed from viral RNA using a BioRad iCycler™ (BioRad, Hercules, CA, USA) thermal cycler as described previously [27].

The generation of a recombinant F-MuLV encoding the bright fluorescent protein mWasabi (wF-MuLV) has been described previously [28]. Briefly, the green fluorescent protein mWasabi [29] was fused to the C-terminus of the F-MuLV envelope, using the 2A self-cleaving peptide of porcine teschovirus for the joining of the sequences [30] (Figure S1, Supplementary Materials). Cloning was performed using the plasmid pFB29 that encodes a permuted clone of F-MuLV strain FB29 [31] (kindly provided by Dr. Marc Sitbon, Institut Génétique Moléculaire de Montpellier, Montpellier, France; kindly transferred by Dr. Masaaki Miyazawa, Kindai University Faculty of Medicine, Osaka, Japan). A ClaI-AscI fragment containing part of F-MuLV Env p15E, a glycine-serine linker, mWasabi, and F-MuLV U3 was synthesized (GeneArt, ThermoFisher, Regensburg, Germany) and subcloned into pBluescript; the 2A sequence was assembled from oligonucleotides (Biomers, Ulm, Germany) and inserted between the glycine-serine linker and the mWasabi coding sequence. The resulting ClaI-AscI fragment containing the C-terminus of p15E, 2A peptide, mWasabi, and U3 was introduced into pFB29 with ClaI and AscI. For reconstitution of the mWasabi-encoding F-MuLV (wF-MuLV), the genome was released from the pFB29-2A-mWasabi plasmid by HindIII digestion, religated and transfected into 293T cells. Recovered virus was purified from supernatants of transfected 293T cells, passaged on *Mus dunni* cells, and virus stocks were prepared as described above.

IgG-opsonization of F-MuLV (F-MuLV-IgG) was done by incubation of the virus with 5 μg/mL, 0.5 μg/mL, or 0.05 μg/mL of FV envelope-specific non-neutralizing monoclonal antibody clone 48 [32] for 60 min at 37 °C. F-MuLV was also opsonized in the presence of normal mouse serum (NMS) as source of complement at a dilution of 1:10 for 60 min at 37 °C (F-MuLV-C). As controls, F-MuLV incubated in medium alone or in heat-inactivated NMS (F-MuLV) was used. After opsonization to remove NMS and unbound IgG, the virus was ultracentrifuged (23,000 × *g*, 2 h, 4 °C) and the virus pellet was resuspended in RPMI. To prove opsonization patterns a virus capture assay (VCA) was routinely performed. Briefly, a 96-well ELISA plate was coated with rabbit anti-mouse IgG or rabbit anti-mouse C3 antibodies. An equal amount of viral RNA according to RT-PCR results from differentially opsonized virus stocks was added and the plate was incubated overnight at 4 °C. Then unbound virus was removed by washing and RNA from bound virus was determined by RT-PCR.

### 2.3. Generation of Bone Marrow-Derived Dendritic Cells

Bone marrow-derived DCs (bmDCs) were generated as described previously [33]. Briefly, 2 × 10^6^ bone marrow cells isolated from femurs and tibiae of female C57BL/6 wt, FcγRI^−/−^, FcγRII^−/−^ and FcγRIII^−/−^ mice were cultivated in 10 mL of RPMI 1640 supplemented with 10% FCS, 2 mM l-glutamine, 500 nM 2-ME and 4 ng/mL of recombinant mouse GM-CSF and recombinant mouse IL-4 (BD Pharmingen, San Jose, CA, USA) for 3 days. A 10 mL portion of fresh medium supplemented with GM-CSF and IL-4 was then added and cells were cultured for another 3 days. On day 6, non-adherent cells were collected, washed and cultivated in fresh 20 mL medium supplemented with GM-CSF and IL-4. On day 8, loosely adherent differentiated bmDCs were harvested and used for experiments. The phenotype of differentiated bmDC cultures was routinely checked by FACS referring to a myeloid DC phenotype (>85% CD11c and >95% CD11b) in bmDC cultures.

### 2.4. Isolation of CD8 T Cells and B Cells

FV-specific and OVA-specific CD8 T cells were isolated from the spleens of female FV-specific CD8 TCR tg and OVA-specific TCR tg OT-1 mice using the BD IMag CD8 T Lymphocyte Enrichment Set (BD Pharmingen) according to the manufacturer’s instructions (purity >95% as determined by FACS). Splenic B cells were isolated from the spleen of female C57BL/6 wt, FcγRI^−/−^, FcγRII^−/−^, and FcγRIII^−/−^ mice using a mouse B cell isolation kit (Miltenyi Biotech, Bergisch Gladbach, Germany) according to the manufacturer’s instructions. Isolated B cell purity was more than 95%, as determined by flow cytometry.

### 2.5. F-MuLV Infection of bmDCs

For this study, 5 × 10^5^ bmDCs were infected with 5000 FFUs of F-MuLV or an equivalent of F-MuLV-C or F-MuLV-IgG based on viral RNA content. After overnight incubation, the input virus was removed and cells were cultivated for 5 days at 37 °C. Supernatants were collected after 24 h and 5 days of culture and applied in ICA to determine productive infection. Alternatively, 5 × 10^5^ bmDCs were infected with 5000 FFUs of wF-MuLV in the presence or absence of clone 48 antibody (Ab) or isotype control Ab (IgG2a) and incubated for 5 days. Wasabi-positive infected cells were then determined by flow cytometry. 

### 2.6. Coculture of FV- or OVA-Loaded bmDCs with Specific CD8 T Cells

DCs were generated from C57BL/6 wt, FcγRI^−/−^, FcγRII^−/−^, and FcγRIII^−/−^ mice. Subsequently, 5 × 10^5^ bmDCs were loaded with 5000 FFUs of F-MuLV or an equivalent of F-MuLV-IgG based on viral RNA content and incubated overnight at 37 °C. Then, the input virus was removed by washing and bmDCs were cocultured with 5 × 10^5^ isolated FV-specific CD8 TCR tg T cells for 48 h. Alternatively, ovalbumin (OVA) (0.1, 1, 10, and 100 μg/mL) was preincubated for 10 min either alone or in the presence of ovalbumin-specific monoclonal Abs (1, 10, and 100 μg/mL from Abcam, Cambridge, UK) to generate OVA-ICs. OVA or OVA-ICs were then loaded to 5 × 10^5^ bmDCs and incubated overnight at 37 °C. Then, bmDCs were washed and cocultured with 5 × 10^5^ isolated OVA-specific CD8 TCR tg OT-1 T cells. After 48 hours of co-cultivation at 37 °C, CD8 T cells were analyzed for the expression of activation markers CD25 and CD69 by FACS. Coculture of F-MuLV-loaded spleen B cells derived from C57BL/6 wt, FcγRI^−/−^, FcγRII^−/−^, and FcγRIII^−/−^ mice was performed as described previously [34]. Briefly, 1 × 10^6^ splenic B cells were loaded with virus and subsequently cocultured with 1 × 10^6^ isolated FV-specific CD8 TCR tg T cells for 48 h. Again, CD8 T cells were analyzed for the expression of activation markers CD25 and CD69 using FACS.

## 3. Results

### 3.1. IgG-Opsonization of F-MuLV Abrogates the Capacity of bmDCs but not B Cells to Activate FV-Specific CD8 T Cells

**Figure 1:**
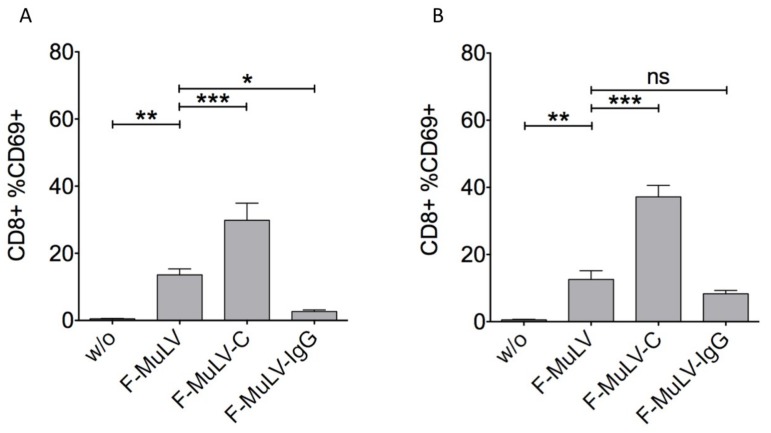
IgG-opsonization of Friend murine leukemia virus (F-MuLV) diminishes activation of virus-specific CD8 T cells by bone marrow-derived dendritic cells (bmDCs) but not by B cells. 5 × 10^5^ (**A**) DCs or (**B**) B cells were loaded with 5000 FFUs of F-MuLV or an equivalent of C’- or IgG-opsonized F-MuLV (F-MuLV-C or F-MuLV-IgG, respectively) based on viral RNA. DCs or B cells were cocultured with 5 × 10^5^ isolated FV-specific TCRtg CD8 T cells. Activation of CD8 T cells was determined by the expression of the early activation marker CD69 gating on AAD-negative living CD8 singlets measured by flow cytometry after 48 h of coculture. Bars represent mean ± SEM of five independent experiments. Data were analyzed by GraphPad PRISM (version 7) software (GraphPad Software, San Diego, CA, USA) using ANOVA followed by Dunnet’s multiple comparison test (***, **, * significant at *p* < 0.001, *p* < 0.01, *p* < 0.05, respectively).

Previous studies suggest that the opsonization pattern on the surface of retroviral particles has an impact on DC-mediated activation of retrovirus-specific CD8 T cells [27,34]. Thus, we investigated the capacity of DCs and B cells infected with differentially opsonized F-MuLV to activate FV-specific CD8 T cells. To this end, F-MuLV was opsonized in the presence of either NMS as source of complement or F-MuLV env-specific Abs. The opsonization pattern was verified in a virus capture assay (VCA) demonstrating the presence of C3 fragments on complement-opsonized (F-MuLV-C) and IgG molecules on Ab-opsonized virus (F-MuLV-IgG) (Figure S2, Supplementary Materials). Bone marrow-derived DCs or isolated spleen B cells were loaded with 5000 FFUs of F-MuLV or an equivalent of differentially opsonized F-MuLV based on viral RNA content. Then, they were cocultured with naïve FV-specific TCRtg CD8 T cells specific for the FV GagL epitope. Activation of CD8 T cells was determined by the expression of the early activation marker CD69 measured by flow cytometry after 48 hours of coculture. According to our previous observations, both DCs (Figure 1A) and B cells (Figure 1B) infected with F-MuLV were able to activate specific CD8 T cells and this activation was significantly enhanced if F-MuLV was opsonized with complement (Figure 1, F-MuLV vs. F-MuLV-C). Interestingly, IgG-opsonization of F-MuLV resulted in a different outcome in T cell activation depending on the antigen-presenting cell involved. Whereas IgG-opsonization of F-MuLV significantly abrogated DC-mediated activation of specific CD8 T cells, it did not affect B cell-mediated activation of FV-specific TCRtg CD8 T cells (Figure 1, F-MuLV vs. F-MuLV-IgG).

### 3.2. IgG-Opsonization Diminishes F-MuLV Infection of DCs

**Figure 2:**
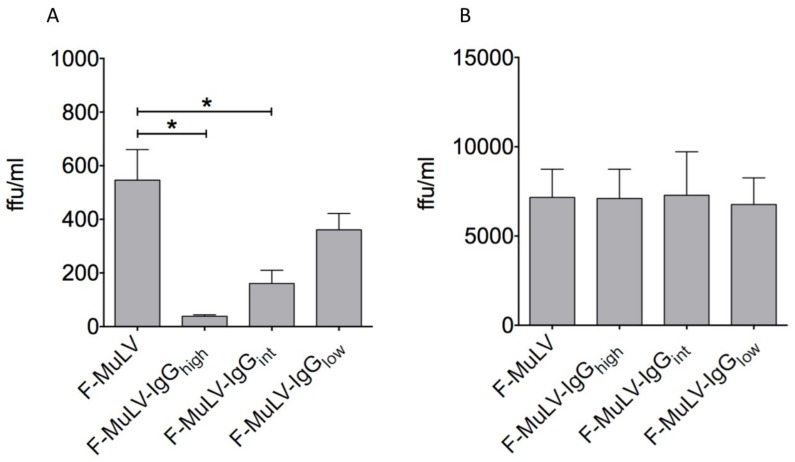
IgG-opsonization diminishes F-MuLV infection of DCs. F-MuLV stocks were opsonized in the presence of 5 μg/mL (F-MuLV-IgGhigh), 0.5 μg/mL (F-MuLV-IgGint), or 0.05 μg/mL (F-MuLV-IgGlow) FV-specific IgG molecules. (**A**) DCs or (**B**) *Mus dunni* cells were infected with 5000 FFUs of F-MuLV or IgG-opsonized F-MuLV. After overnight incubation, the input virus was removed by washing and cells were further cultivated up to 5 days at 37 °C. Supernatants were collected after 24 h and 5 days of culture and applied in an infectious center assay to determine productive infection. Data represent mean ± SEM of two independent experiments. Data were analyzed by GraphPad PRISM software using ANOVA followed by Dunnet’s multiple comparison test (***, **, * significant at *p* < 0.001, *p* < 0.01, *p* < 0.05, respectively).

As complement-mediated enhancement of specific CD8 T cell activation by DCs was accompanied with an enhanced infection of DC by F-MuLV-C [27], we next analyzed the impact of IgG-opsonization of F-MuLV on DC infection levels. We generated F-MuLV stocks opsonized in the presence of 5 μg/mL, 0.5 μg/mL, or 0.05 μg/mL FV-specific IgG molecules resulting in virus stocks with relatively high (F-MuLV-IgGhigh), intermediate (F-MuLV-IgGint) or low (F-MuLV-IgGlow) quantities of IgG molecules bound to the viral surface as demonstrated in VCA (Figure S2B, Supplementary Materials). DCs were infected with 5000 FFUs of F-MuLV or an equivalent of F-MuLV-IgG based on viral RNA content. The input virus was removed by washing and virus titers in supernatants from 5-day cultures were determined using permissive *Mus dunni* cells in an infectious center assay. IgG-opsonization of F-MuLV reduced productive infection of DCs and the level of reduction was dependent on the IgG concentration used for opsonization (Figure 2A). Compared to F-MuLV, the infection of DCs was significantly reduced if infected with F-MuLV-IgGhigh or F-MuLV-IgGint (Figure 2A). In contrast, FcγR non-expressing *Mus dunni* cells showed similar infection from both F-MuLV and IgG-opsonized F-MuLV, which excludes a potential neutralization by the Abs and suggests an FcγR-mediated effect on the level of infection (Figure 2B).

We confirmed this data using wF-MuLV, a F-MuLV encoding the fluorescent protein mWasabi, to infect bmDCs in the presence or absence of different concentrations of the FV-specific Ab clone 48 (from 5 to 0.0005 μg/mL). The presence of the FV-specific Ab inhibited the F-MuLV infection of bmDCs in a concentration-dependent manner (Figure S3A, Supplementary Materials), whereby we could detect a significant reduction of DC infection when using clone 48 at a concentration of 5 μg/mL. In contrast, isotype control Ab (5 μg/mL) or lower concentrations (0.5–0.0005 μg/mL) of clone 48 did not significantly affect F-MuLV infection of DCs.

### 3.3. Abrogation of the Capacity of DCs to Activate FV-Specific CD8 T Cells Correlates with the Diminished Infection of DCs by IgG-Opsonized F-MuLV

**Figure 3:**
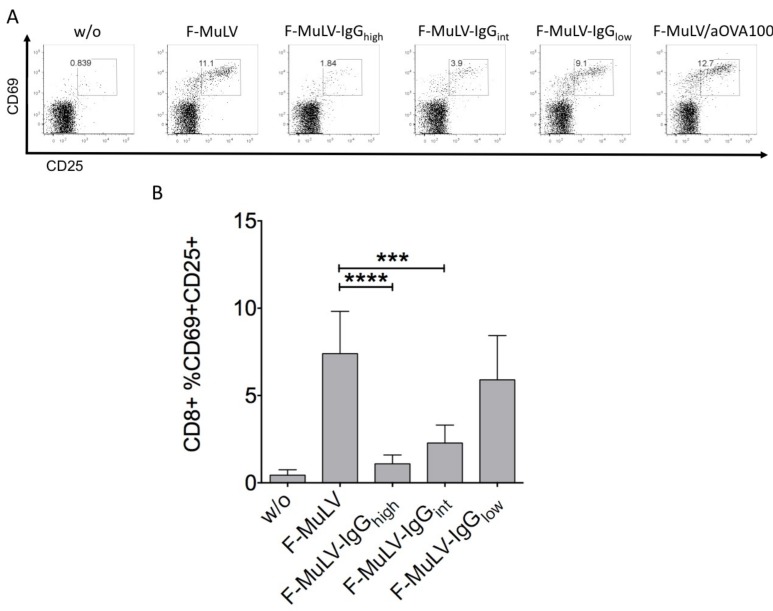
Reduction of CD8 T cell activation was dependent on the concentration of IgG molecules used for F-MuLV opsonization. F-MuLV stocks were opsonized in the presence of 5 μg/mL (F-MuLV-IgG_high_), 0.5 μg/mL (F-MuLV-IgG_int_), or 0.05 μg/mL (F-MuLV-IgG_low_) FV-specific IgG molecules. As a control, F-MuLV was opsonized in the presence of 100 μg/mL OVA-specific Abs (F-MuLV/aOVA100). 5 × 10^5^ DCs were loaded with 5000 FFUs of F-MuLV or IgG-opsonized F-MuLV and cocultured with 5 × 10^5^ isolated FV-specific TCRtg CD8 T cells. Activation of CD8 T cells was determined by the expression of CD25 and the early activation marker CD69 gating on AAD-negative living CD8 singlets measured by flow cytometry after 48 hours of coculture. (**A**) A representative experiment and (**B**) mean ± SEM derived from five independent experiments are shown. Data were analyzed by GraphPad PRISM software using ANOVA followed by Dunnet’s multiple comparison test (***, **, * significant at *p* < 0.001, *p* < 0.01, *p* < 0.05, respectively).

To further characterize the effect of IgG-opsonization on DC-mediated activation of specific CD8 T cells, F-MuLV stocks opsonized in the presence of 5 μg/mL, 0.5 μg/mL, or 0.05 μg/mL FV-specific IgG molecules, resulting in virus stocks with relatively high (F-MuLV-IgG_high_), intermediate (F-MuLV-IgG_int_), or low (F-MuLV-IgG_low_) quantities of IgG molecules bound to the viral surface, were used. In coculture experiments with virus-loaded DCs and naïve FV-specific CD8 T cells, we found that the level of the reduction in CD8 T cell activation was dependent on the concentration of IgG molecules used for F-MuLV opsonization only resulting in a significant reduction of CD8 T cell activation if F-MuLV-IgG_high_ or F-MuLV-IgGint were used (Figure 3B). The effect of IgG-opsonization on CD8 T cell activation was related to a specific recognition of the FV envelope by the Abs, as we did not detect any reduction of the CD8 T cell activation if F-MuLV was incubated in the presence of 100 μg/mL OVA-specific Abs (Figure 3A, F-MuLV vs. F-MuLV/aOVA100).

We again confirmed the above results using bmDCs infected with 5000 FFUs of wF-MuLV in the presence or absence of different concentrations of FV-specific IgG (from 5 to 0.0005 μg/mL clone 48 mAb). As a control, bmDCs were infected in the presence of 5 μg/mL isotype control Ab. Infected DCs were subsequently cocultured with FV-specific CD8 T cells. As above, we found a significant reduction in the capacity of DCs to activate virus-specific CD8 T cells if 5 μg/mL clone 48 were present during DC infection (Figure S3B, Supplementary Materials). In contrast, neither isotype control Ab (5 μg/mL) nor lower concentrations of clone 48 mAb (0.5–0.0005 μg/mL) resulted in significant changes in CD8 T cell activation (Figure S3B, Supplementary Materials).

### 3.4. In contrast to F-MuLV-IgG, Immune-Complexed IgG-OVA Facilitates DC-Mediated Activation of OVA-Specific OT-1 CD8 T Cells

**Figure 4:**
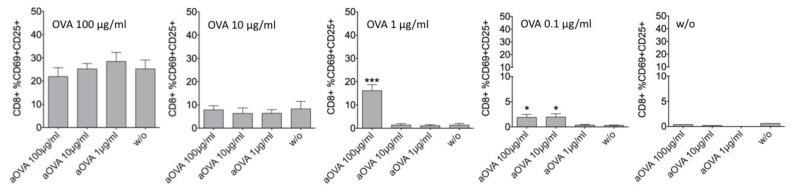
Immune-complexed IgG-OVA facilitates DC-mediated activation of OVA-specific OT-1 CD8 T cells. Ovalbumin (0.1, 1, 10, and 100 μg/mL) was preincubated for 10 min either alone or in the presence of ovalbumin-specific Abs (1, 10, and 100 μg/mL) to generate OVA-ICs. OVA or OVA-ICs were then loaded to 5 × 10^5^ bmDCs and incubated overnight at 37 °C. Then, bmDCs were washed and cocultured with 5 × 10^5^ isolated OVA-specific CD8 TCR tg OT-1 T cells for 48 h. After 48 h of co-cultivation, activation of CD8 T cells was determined by the expression of CD25 and the early activation marker CD69 gating on AAD-negative living CD8 singlets measured by FACS. Data represent mean ± SEM of three independent experiments. Data were analyzed by GraphPad PRISM software using ANOVA followed by Dunnet’s multiple comparison test (***, **, * significant at *p* < 0.001, *p* < 0.01, *p* < 0.05, respectively).

Our findings with F-MuLV immune-complexed with specific IgGs are contradicting several previous studies demonstrating FcγR-mediated enhancement of CD8 T cell responses by DCs if using immune-complexed Ags. Thus, we tested whether bmDCs loaded with OVA-ICs could facilitate the activation of OVA-specific OT-1 CD8 T cells using our experimental settings. Indeed, OVA immune complexes generated with OVA-specific Abs were able to facilitate antigen presentation because we detected significantly enhanced DC-mediated activation of OVA-specific OT-1 cells with 1 μg/mL OVA in the presence of 100 μg/mL OVA-specific Abs as well as with 0.1 μg/mL OVA in the presence of 10 or 100 μg/mL OVA-specific Abs (Figure 4, OVA 1 μg/mL and 0.1 μg/mL). This enhancement was dependent on the generation of OVA-ICs as no enhancement was observed using OVA and non-specific control Abs (Figure S4, Supplementary Materials, OVA1 vs. OVA1 + iso100). The fact that no enhancement of OVA-specific OT-1 CD8 T cells was seen at higher OVA concentrations (Figure 4, OVA 100 μg/mL and 10 μg/mL) suggests a requirement of at least 3 to 30 times molar excess of anti-OVA (aOVA) over OVA to efficiently trigger CD8 T cell activation. These data suggest differences in handling between protein Ag and living virus containing ICs by DCs with respect to the subsequent antigen processing/presentation.

### 3.5. Impairment of Specific CD8 T Cell Activation by F-MuLV-IgG Loaded DCs is Mediated by FcγRI (CD64)

**Figure 5:**
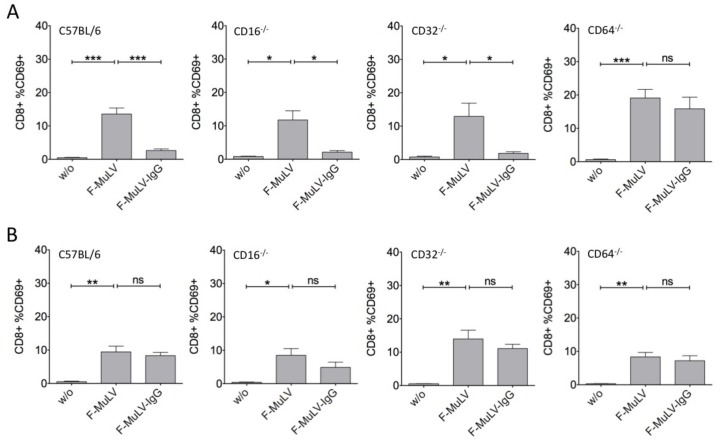
Impairment of specific CD8 T cell activation by F-MuLV-IgG loaded DCs is mediated by Fcγ receptor type I (FcγRI) (CD64). 5 × 10^5^ (**A**) bmDCs or (**B**) B cells derived from C57BL/6 wt or from FcγRI (CD64^−/−^), FcγRII (CD32^−/−^), or FcγRIII (CD16^−/−^)-deficient mice were loaded with 5000 FFUs of F-MuLV or IgG-opsonized F-MuLV (F-MuLV-IgG) and cocultured with 5 × 10^5^ isolated FV-specific TCRtg CD8 T cells. Activation of CD8 T cells was determined by the expression of the early activation marker CD69 gating on AAD-negative living CD8 singlets measured by flow cytometry after 48 h of coculture. Data represent mean ± SEM of three or four independent experiments. Data were analyzed by GraphPad PRISM software using ANOVA followed by Dunnet’s multiple comparison test (***, **, * significant at *p* < 0.001, *p* < 0.01, *p* < 0.05, respectively).

IgG-mediated effects on CD8 T cell activation by DCs have been reported to be associated with the interaction of Ag-ICs with FcγRs expressed on DCs. As bone marrow-derived DCs express activating FcγRI, FcγRIII, and FcγRIV as well as inhibitory FcγRIIb, we analyzed the role of FcγRs in the opsonized virus-mediated abrogation of CD8 T cell activation by using DCs generated from mice deficient for FcγRI, FcγRII, or FcγRIII. DCs derived from different FcγR-deficient mice were loaded with F-MuLV or F-MuLV-IgG and subsequently cells were cocultured with FV-specific CD8 TCRtg T cells. Similar to wt bmDCs (Figure 5A, C57BL/6), IgG-opsonization of F-MuLV significantly reduced the capacity of DCs to activate specific CD8 T cells if DCs were generated from the bone marrow of FcγRIII (Figure 5A, CD16^−/−^) or FcγRII (Figure 5A, CD32^−/−^)-deficient mice. However, IgG-mediated reduction in the capacity of DCs to activate specific T cells was alleviated when DCs were derived from FcγRI (Figure 5A, CD64^−/−^)-deficient mice. We also performed coculture experiments of FV CD8 TCRtg T cells with B cells isolated from spleen of wt and different FcγR-deficient mice. B cells are thought to express only inhibitory FcγRII. Similar to wt B cells, none of the B cells isolated from different FcγR-deficient mice showed significant differences in their capacity to activate FV-specific CD8 TCRtg T cells if loaded with F-MuLV or F-MuLV-IgG (Figure 5B). These results suggest a crucial role of CD64 in IgG-mediated abrogation of CD8 T cell activation by DCs.

**Figure 6:**
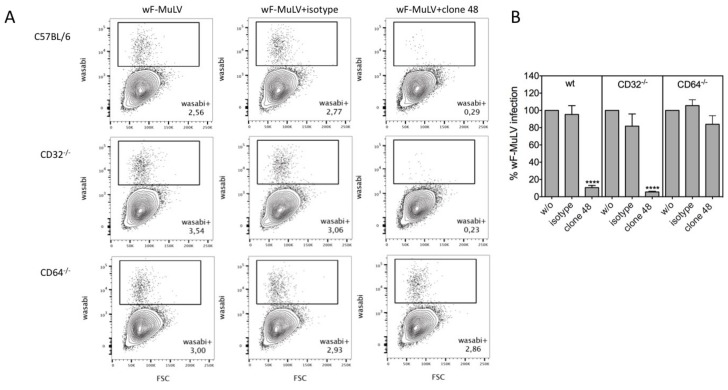
Abrogated infection by IgG-opsonized F-MuLV is restored in CD64-deficient DCs. DCs derived from the bone marrow of C57BL/6 wt or FcγRI (CD64^−/−^) or FcγRII (CD32^−/−^)-deficient mice were infected with 5000 FFUs of mWasabi-encoding F-MuLV (wF-MuLV) in the presence or absence of clone 48 or isotype antibodies. Cells were further cultivated for 2 days at 37 °C and mWasabi-positive infected cells were determined by FACS. (**A**) Density plots showing mWasabi-positive cells from a representative experiment and (**B**) data representing mean ± SEM of four independent experiments are shown (activation measured in control samples (*w*/*o*) was set to 100%). Data were analyzed by GraphPad PRISM software using ANOVA followed by Sidak’s multiple comparison test.

Since the reduction in the capacity of DCs to activate virus-specific CD8 T cells correlated with the impaired infection of DCs by IgG-opsonized F-MuLV (Figure 2B and Figure 3A), we repeated infection experiments with DCs derived from wt and FcγR-deficient mice using a F-MuLV encoding the fluorescent protein mWasabi (wF-MuLV). Corresponding to the impaired capacity to activate CD8 T cells in cocultures with F-MuLV-IgG, both wt and FcγRII-deficient bmDCs showed a significantly reduced wF-MuLV infection in the presence of virus-specific Abs (Figure 6A,B). In contrast, F-MuLV infection was not influenced by IgG-opsonization in FcγRI-deficient bmDCs suggesting a pivotal role of CD64 for IgG-mediated effects on retroviral infection of DCs.

## 4. Discussion

In this study, we demonstrated that IgG-opsonization of F-MuLV abrogated the activation of FV-specific CD8 T cells by DCs, which correlated with impaired infection of DCs. Using bmDCs derived from FcγR-deficient mice, we showed that IgG-opsonization abolished DC-mediated activation of specific CD8 T cells via FcγRI.

Retroviruses like HIV evolved mechanisms to escape from the destruction of complement-mediated lysis even though they activate the complement system [35]. This complement activation and insufficient lysis result in a deposition of complement fragments on the viral surface. After seroconversion antibodies also cover viral particles in vivo, which can be experimentally recapitulated in vitro. The opsonization pattern on retroviral particles (e.g., the presence of C3-fragments and/or IgGs) has been shown to have an impact on the capacity of human as well as mouse DCs to activate specific CTLs [27]. Using FV, a mouse retrovirus model, we further investigated the capacity of DCs and B cells loaded with differentially opsonized F-MuLV to induce FV-specific CD8 T cell activation. In line with our previous observations, both DCs and B cells significantly increased the activation of FV-specific CD8 T cells with C’-opsonized virus (F-MuLV-C) when compared to non-opsonized controls (F-MuLV) [27,34]. In contrast to this, we observed a significantly decreased activation of specific CD8 T cells using DCs incubated with IgG-opsonized F-MuLV (F-MuLV-IgG) when compared to F-MuLV. In previous studies, C’ opsonization of HIV increased the activation of virus-specific CD8 T cells; however, HIV-specific IgG molecules deposited on the viral surface did not significantly affect CD8 T cell activation compared to non-opsonized HIV [16]. IgG-opsonization however clearly reduced C’-mediated enhancement of CD8 T cell activation induced by virus-loaded DCs [16]. IgG-opsonization of F-MuLV did not influence CD8 T cell activation by B cells suggesting that this difference in IgG-mediated effects seen between DCs and B cells might be related to a differential expression of FcγRs on these cells. Interestingly, our observation is in conflict with previous investigations in which FcγRs have been shown to facilitate activation of both specific CD8 and CD4 T cells by DCs if protein/Ab complexes were used [3,6,11,12,13,14]. We confirmed these findings for CD8 T cell activation with OVA/Ab complexes. This discrepancy might be explained by the difference in immune-complexed Ags used. Whereas in our study immune-complexed infectious viral particles were used, studies investigating the involvement of FcγRs in T cell activation were performed mainly with soluble protein antigens. 

Since the infection of APCs can influence the presentation of Ags in an MHC class I context, we investigated the infectivity of IgG-opsonized F-MuLV in bmDC. Compared to non-opsonized F-MuLV, in line with previous observations, C’ opsonization significantly increased infection of both DCs and B cells [27,34]. Interestingly, infection of DCs was significantly reduced if the virus was opsonized in the presence of FV-specific Abs, similar to what has been found in human DCs with IgG-opsonized HIV [15]. In contrast to these data, FcγRs are not able to influence retroviral infection of human CD4 T cells with immune-complexed HIV [36]. Moreover, adenoviral transduction of DCs is enhanced by FcγRI targeting [37]. Such antibody-dependent enhancement (ADE) of infection has been reported in various viral infections, and ADE is particularly important in dengue virus (DENV) infections [38,39]. ADE primarily occurs if virus-specific antibodies aid infection of immune-complexed viruses through FcγRs. Several factors, such as the infecting virus strain, the antibody concentration, and the epitope availability, have all been demonstrated to be involved in FcR-dependent ADE [38]. As suboptimal antibody concentrations have been shown to play a role in ADE of DENV infections, we also performed DC infection and subsequent coculture experiments in the presence of FV-specific antibodies ranging from 5 to 0.0005 μg/mL. In our experimental setting in vitro using bmDCs, we did not find any direct ADE of F-MuLV. To our knowledge, no ADE has been reported in the Friend virus model, which is in line with our data. However, ADE of viral infections involves several factors not investigated in our present study. F-MuLV infection of myeloid cells other than DCs like monocytes/macrophages might be different if using IgG-opsonized virus. Furthermore, IgG-opsonization of viruses through the impairment of DC-mediated activation of virus-specific CD8 T cell responses might be indirectly involved in ADE, but this needs to be further investigated. Although FcγRs have been reported to enhance internalization of immune-complexed Ags [10], their interaction with IgG molecules on the viral surface inhibits retroviral infection. We could exclude an abrogated infection due to a putative neutralizing capacity of FV-specific Abs, since FV envelope-specific monoclonal antibody clone 48 does not neutralize FV in vitro [32,40] and in line with this observation, the infection of FV permissive *Mus dunni* cells with differentially opsonized FV stocks was similar in our study. Of note, *Mus dunni* cells express neither FcγRs nor CRs on their surface. Differential expression pattern of FcγRs on DCs and B cells also supports an involvement of FcγRs in the abrogation of FV infection of DCs rather than neutralization by Abs [41]. Finally, infection experiments with wF-MuLV in wt and FcγR-deficient DCs clearly demonstrated that IgG abrogated DC infection solely through the interaction of IgG-opsonized virus with CD64.

IgG-mediated effects on infection and antigen presentation depend most likely on FcγRs on DCs. Therefore, to further investigate the particular FcγR on DCs influencing infection and APC functions, we utilized DCs and B cells derived from FcγR-deficient mice. Using bmDCs derived from FcγRI, RII, and RIII knockout mice, we found restored capacity of DC-loaded IgG-opsonized FV to activate FV-specific CD8 T cells if DCs were generated from FcγRI KO mice. These results point to an involvement of CD64 in the inhibition of FV-specific CD8 T cell activation by DCs. It is puzzling that an activating receptor (CD64) reduces Ag presentation. Binding of immune-complexed F-MuLV to DCs might induce signaling events resulting in a change in DC functions related to a less efficient CD8 T cell activation. A study on the human respiratory syncytial virus (hRSV) shows that IgG-opsonization of the virus leads to an abortive infection of DCs resulting in an impaired T cell activation by DCs [42]. However, this effect was mediated by FcγRs, an involvement of either cytokines like IL-10 and IL-12 or DC maturation has not been demonstrated [42]. Viruses among other pathogens can be sensed by DCs through pattern recognition receptors like Toll-like receptors (TLR). Thus, a cross-talk between signaling pathways induced by FcγRs and TLRs might also impact antigen presentation by DCs [43]. In human macrophages, the stimulation of FcγRs leads to a decreased HIV infection related to an inhibition of proviral integration [44]. Thus, IgG-opsonization through CD64 might interfere with intrinsic retroviral restriction pathways in DCs known to be involved in FV infections [45,46,47], similar to what is shown for complement-opsonization in HIV infection of human DCs [48].

In the human system, abrogated infection and diminished CD8 T cell activation has been found when DCs were loaded with HIV-ICs [15]. In contrast to our results in mice, inhibitory FcγRIIb has been shown to be responsible for the impaired Ag-presenting capacity of DCs, whereas activating FcγRIIa was able to enhance infection [15]. Of note, human monocyte-derived DCs used in this study express exclusively FcγRIIa and FcγRIIb on their surface. Other studies reported a role of CD64 in the enhancement of CD8 T cell response in mice. It has been shown that OVA-ICs are much more efficiently presented for CD8 T cells than soluble OVA in an FcγR-dependent manner [3]. Cross-presentation of immune-complexed Ags by DCs was also mediated through activating FcγRs (FcγRI and FcγRIII), and no enhancement of MHC class I-restricted Ag presentation was observed by DCs from γ-chain-deficient mice [7,13]. Nevertheless, in the human system, IgG-opsonization of HIV directed the virus into MHC class II-associated compartments, whereas non-opsonized and C’-opsonized virions were mainly associated with MHC class I compartments, suggesting that IgG-opsonization is rather supporting MHC class II presentation than direct CTL activation [15].

Conflicting data regarding the role of inhibitory FcγRII in the regulation of Ag presentation have been reported before. Inhibitory FcγRII is thought to diminish cellular responses when cross-linked with activating FcγRs [1]. FcγRII inhibited CD8 T cell induction through the suppression of both DC activation and cross-presentation of Ags by activating FcγRs [14]. In contrast, other studies indicate an improvement of uptake and presentation of IC by DCs through FcγRII [3,13]. However, in our study, we did not observe any influence of FcγRII in CTL activation by both DCs or B cells loaded with immune-complexed FV.

The conflicting data on the influence of different FcγRs on CD8 T cell responses might be explained by the usage of different Ags. Although obviously mediated through different FcγRs in humans and mice, infectivity of immune-complexed viruses can be reduced by their binding to FcγRs on DCs. However, differences in FcγR expression pattern on different immune cell populations allow only a careful extrapolation of findings in mice to humans. Furthermore, in vitro experiments using defined APC populations might provide different results compared to in vivo data where the interplay of different APCs expressing different FcγR patterns on their surface makes the situation more complex. Whether the abrogated infection of DCs by immune-complexed FV through CD64 is dependent on different intracellular trafficking (e.g., elimination of viruses in endosomal compartments) or other cellular mechanisms (e.g., cellular signaling pathways or inhibition of the integration of the proviral genome) needs further investigation.

## Supplementary Materials

The following are available online at http://www.mdpi.com/1999-4915/11/2/145/s1, Figure S1: Schematic representation of the mWasabi-encoding F-MuLV, Figure S2: VCA demonstrates the presence of (A) C3 fragments on complement-opsonized (F-MuLV-C) and (B) IgG molecules on Ab-opsonized (F-MuLV-IgG) virus, Figure S3: FV-specific IgG-mediated, concentration-dependent abrogation of bmDC infection by wF-MuLV correlates with decreased capacity of DCs to activate virus-specific CD8 T cells, Figure S4: Immune-complexed IgG-OVA facilitates DC-mediated activation of OVA-specific OT-1 CD8 T cells.

## References

[1] Amigorena S., Bonnerot C. (1999). Fc receptor signaling and trafficking: A connection for antigen processing. Immunol. Rev..

[2] Bajtay Z., Csomor E., Sandor N., Erdei A. (2006). Expression and role of Fc- and complement-receptors on human dendritic cells. Immunol. Lett..

[3] De Jong J.M., Schuurhuis D.H., Ioan-Facsinay A., van der Voort E.I., Huizinga T.W., Ossendorp F., Toes R.E., Verbeek J.S. (2006). Murine Fc receptors for IgG are redundant in facilitating presentation of immune complex derived antigen to CD8+ T cells in vivo. Mol. Immunol..

[4] McKenzie S.E., Taylor S.M., Malladi P., Yuhan H., Cassel D.L., Chien P., Schwartz E., Schreiber A.D., Surrey S., Reilly M.P. (1999). The role of the human Fc receptor Fc gamma RIIA in the immune clearance of platelets: A transgenic mouse model. J. Immunol..

[5] Ravetch J.V., Bolland S. (2001). IgG Fc receptors. Annu. Rev. Immunol..

[6] Den Haan J.M., Bevan M.J. (2002). Constitutive versus activation-dependent cross-presentation of immune complexes by CD8(+) and CD8(−) dendritic cells in vivo. J. Exp. Med..

[7] Regnault A., Lankar D., Lacabanne V., Rodriguez A., Thery C., Rescigno M., Saito T., Verbeek S., Bonnerot C., Ricciardi-Castagnoli P. (1999). Fcgamma receptor-mediated induction of dendritic cell maturation and major histocompatibility complex class I-restricted antigen presentation after immune complex internalization. J. Exp. Med..

[8] Banki Z., Kacani L., Mullauer B., Wilflingseder D., Obermoser G., Niederegger H., Schennach H., Sprinzl G.M., Sepp N., Erdei A. (2003). Cross-linking of CD32 induces maturation of human monocyte-derived dendritic cells via NF-kappa B signaling pathway. J. Immunol..

[9] Boruchov A.M., Heller G., Veri M.C., Bonvini E., Ravetch J.V., Young J.W. (2005). Activating and inhibitory IgG Fc receptors on human DCs mediate opposing functions. J. Clin. Investig..

[10] Manca F., Fenoglio D., Li Pira G., Kunkl A., Celada F. (1991). Effect of antigen/antibody ratio on macrophage uptake, processing, and presentation to T cells of antigen complexed with polyclonal antibodies. J. Exp. Med..

[11] De Jong J.M., Schuurhuis D.H., Ioan-Facsinay A., Welling M.M., Camps M.G., van der Voort E.I., Huizinga T.W., Ossendorp F., Verbeek J.S., Toes R.E. (2006). Dendritic cells, but not macrophages or B cells, activate major histocompatibility complex class II-restricted CD4+ T cells upon immune-complex uptake in vivo. Immunology.

[12] Signorino E., Brusa D., Granata R., Malavasi F., Ferrone S., Matera L. (2007). Contribution of dendritic cells’ FcgammaRI and FcgammaRIII to cross-presentation of tumor cells opsonized with the anti-MHC class I monoclonal antibodies. Cancer Biol. Ther..

[13] Yada A., Ebihara S., Matsumura K., Endo S., Maeda T., Nakamura A., Akiyama K., Aiba S., Takai T. (2003). Accelerated antigen presentation and elicitation of humoral response in vivo by FcgammaRIIB- and FcgammaRI/III-mediated immune complex uptake. Cell. Immunol..

[14] Getahun A., Dahlstrom J., Wernersson S., Heyman B. (2004). IgG2a-mediated enhancement of antibody and T cell responses and its relation to inhibitory and activating Fc gamma receptors. J. Immunol..

[15] Wilflingseder D., Banki Z., Garcia E., Pruenster M., Pfister G., Muellauer B., Nikolic D.S., Gassner C., Ammann C.G., Dierich M.P. (2007). IgG opsonization of HIV impedes provirus formation in and infection of dendritic cells and subsequent long-term transfer to T cells. J. Immunol..

[16] Posch W., Cardinaud S., Hamimi C., Fletcher A., Muhlbacher A., Loacker K., Eichberger P., Dierich M.P., Pancino G., Lass-Florl C. (2012). Antibodies attenuate the capacity of dendritic cells to stimulate HIV-specific cytotoxic T lymphocytes. J. Allergy Clin. Immunol..

[17] Hasenkrug K.J., Dittmer U. (2007). Immune control and prevention of chronic Friend retrovirus infection. Front. Biosci..

[18] Hasenkrug K.J., Chougnet C.A., Dittmer U. (2018). Regulatory T cells in retroviral infections. PLoS Pathog..

[19] Drabczyk-Pluta M., Werner T., Hoffmann D., Leng Q., Chen L., Dittmer U., Zelinskyy G. (2017). Granulocytic myeloid-derived suppressor cells suppress virus-specific CD8(+) T cell responses during acute Friend retrovirus infection. Retrovirology.

[20] Teigler J.E., Zelinskyy G., Eller M.A., Bolton D.L., Marovich M., Gordon A.D., Alrubayyi A., Alter G., Robb M.L., Martin J.N. (2017). Differential Inhibitory Receptor Expression on T Cells Delineates Functional Capacities in Chronic Viral Infection. J. Virol..

[21] Dittmer U., Race B., Peterson K.E., Stromnes I.M., Messer R.J., Hasenkrug K.J. (2002). Essential roles for CD8+ T cells and gamma interferon in protection of mice against retrovirus-induced immunosuppression. J. Virol..

[22] Dittmer U., He H., Messer R.J., Schimmer S., Olbrich A.R., Ohlen C., Greenberg P.D., Stromnes I.M., Iwashiro M., Sakaguchi S. (2004). Functional impairment of CD8(+) T cells by regulatory T cells during persistent retroviral infection. Immunity.

[23] Ioan-Facsinay A., de Kimpe S.J., Hellwig S.M., van Lent P.L., Hofhuis F.M., van Ojik H.H., Sedlik C., da Silveira S.A., Gerber J., de Jong Y.F. (2002). FcgammaRI (CD64) contributes substantially to severity of arthritis, hypersensitivity responses, and protection from bacterial infection. Immunity.

[24] Takai T., Ono M., Hikida M., Ohmori H., Ravetch J.V. (1996). Augmented humoral and anaphylactic responses in Fc gamma RII-deficient mice. Nature.

[25] Hazenbos W.L., Heijnen I.A., Meyer D., Hofhuis F.M., Renardel de Lavalette C.R., Schmidt R.E., Capel P.J., van de Winkel J.G., Gessner J.E., van den Berg T.K. (1998). Murine IgG1 complexes trigger immune effector functions predominantly via Fc gamma RIII (CD16). J. Immunol..

[26] Ohlen C., Kalos M., Cheng L.E., Shur A.C., Hong D.J., Carson B.D., Kokot N.C., Lerner C.G., Sather B.D., Huseby E.S. (2002). CD8(+) T cell tolerance to a tumor-associated antigen is maintained at the level of expansion rather than effector function. J. Exp. Med..

[27] Banki Z., Posch W., Ejaz A., Oberhauser V., Willey S., Gassner C., Stoiber H., Dittmer U., Dierich M.P., Hasenkrug K.J. (2010). Complement as an endogenous adjuvant for dendritic cell-mediated induction of retrovirus-specific CTLs. PLoS Pathog..

[28] Windmann S., Otto L., Hrycak C.P., Malyshkina A., Bongard N., David P., Gunzer M., Dittmer U., Bayer W. (2019). Infection of B cell follicle-resident cells by Friend retrovirus occurs during acute infection and is maintained during viral persistence. mBio.

[29] Ai H.W., Olenych S.G., Wong P., Davidson M.W., Campbell R.E. (2008). Hue-shifted monomeric variants of Clavularia cyan fluorescent protein: Identification of the molecular determinants of color and applications in fluorescence imaging. BMC Biol..

[30] Luke G.A., de Felipe P., Lukashev A., Kallioinen S.E., Bruno E.A., Ryan M.D. (2008). Occurrence, function and evolutionary origins of ’2A-like’ sequences in virus genomes. J. Gen. Virol..

[31] Sitbon M., Sola B., Evans L., Nishio J., Hayes S.F., Nathanson K., Garon C.F., Chesebro B. (1986). Hemolytic anemia and erythroleukemia, two distinct pathogenic effects of Friend MuLV: Mapping of the effects to different regions of the viral genome. Cell.

[32] Halemano K., Harper M.S., Guo K., Li S.X., Heilman K.J., Barrett B.S., Santiago M.L. (2013). Humoral immunity in the Friend retrovirus infection model. Immunol. Res..

[33] Lutz M.B., Kukutsch N., Ogilvie A.L., Rossner S., Koch F., Romani N., Schuler G. (1999). An advanced culture method for generating large quantities of highly pure dendritic cells from mouse bone marrow. J. Immunol. Methods.

[34] Bila C., Oberhauser V., Ammann C.G., Ejaz A., Huber G., Schimmer S., Messer R., Pekna M., von Laer D., Dittmer U. (2011). Complement opsonization enhances friend virus infection of B cells and thereby amplifies the virus-specific CD8+ T cell response. J. Virol..

[35] Agrawal P., Nawadkar R., Ojha H., Kumar J., Sahu A. (2017). Complement Evasion Strategies of Viruses: An Overview. Front. Microbiol..

[36] McLain L., Dimmock N.J. (1997). A human CD4+ T-cell line expresses functional CD64 (Fc gamma RI), CD32 (Fc gamma RII), and CD16 (Fc gamma RIII) receptors but these do not enhance the infectivity of HIV-1-IgG complexes. Immunology.

[37] Sapinoro R., Maguire C.A., Burgess A., Dewhurst S. (2007). Enhanced transduction of dendritic cells by FcgammaRI-targeted adenovirus vectors. J. Gene Med..

[38] Taylor A., Foo S.S., Bruzzone R., Dinh L.V., King N.J., Mahalingam S. (2015). Fc receptors in antibody-dependent enhancement of viral infections. Immunol. Rev..

[39] Morens D.M., Halstead S.B. (1990). Measurement of antibody-dependent infection enhancement of four dengue virus serotypes by monoclonal and polyclonal antibodies. J. Gen. Virol..

[40] Chesebro B., Wehrly K., Cloyd M., Britt W., Portis J., Collins J., Nishio J. (1981). Characterization of mouse monoclonal antibodies specific for Friend murine leukemia virus-induced erythroleukemia cells: Friend-specific and FMR-specific antigens. Virology.

[41] DiLillo D.J., Ravetch J.V. (2015). Fc-Receptor Interactions Regulate Both Cytotoxic and Immunomodulatory Therapeutic Antibody Effector Functions. Cancer Immunol. Res..

[42] Gomez R.S., Ramirez B.A., Cespedes P.F., Cautivo K.M., Riquelme S.A., Prado C.E., Gonzalez P.A., Kalergis A.M. (2016). Contribution of Fcgamma receptors to human respiratory syncytial virus pathogenesis and the impairment of T-cell activation by dendritic cells. Immunology.

[43] Van Egmond M., Vidarsson G., Bakema J.E. (2015). Cross-talk between pathogen recognizing Toll-like receptors and immunoglobulin Fc receptors in immunity. Immunol. Rev..

[44] Perez-Bercoff D., David A., Sudry H., Barre-Sinoussi F., Pancino G. (2003). Fcgamma receptor-mediated suppression of human immunodeficiency virus type 1 replication in primary human macrophages. J. Virol..

[45] Takeda E., Tsuji-Kawahara S., Sakamoto M., Langlois M.A., Neuberger M.S., Rada C., Miyazawa M. (2008). Mouse APOBEC3 restricts friend leukemia virus infection and pathogenesis in vivo. J. Virol..

[46] Behrendt R., Schumann T., Gerbaulet A., Nguyen L.A., Schubert N., Alexopoulou D., Berka U., Lienenklaus S., Peschke K., Gibbert K. (2013). Mouse SAMHD1 has antiretroviral activity and suppresses a spontaneous cell-intrinsic antiviral response. Cell Rep..

[47] Li S.X., Barrett B.S., Heilman K.J., Messer R.J., Liberatore R.A., Bieniasz P.D., Kassiotis G., Hasenkrug K.J., Santiago M.L. (2014). Tetherin promotes the innate and adaptive cell-mediated immune response against retrovirus infection in vivo. J. Immunol..

[48] Posch W., Steger M., Knackmuss U., Blatzer M., Baldauf H.M., Doppler W., White T.E., Hortnagl P., Diaz-Griffero F., Lass-Florl C. (2015). Complement-Opsonized HIV-1 Overcomes Restriction in Dendritic Cells. PLoS Pathog..

