# Supplementary material for: Fcγ Receptor Type I (CD64)-Mediated Impairment of the Capacity of Dendritic Cells to Activate Specific CD8 T Cells by IgG-opsonized Friend Virus
Source: Viruses. 2019 Feb 8;11(2):145. doi: 10.3390/v11020145 (PMC6410291; doi:10.3390/v11020145)
Supplement: Supplementary file 1 [file viruses-11-00145-s001.pdf]

Supporting Information

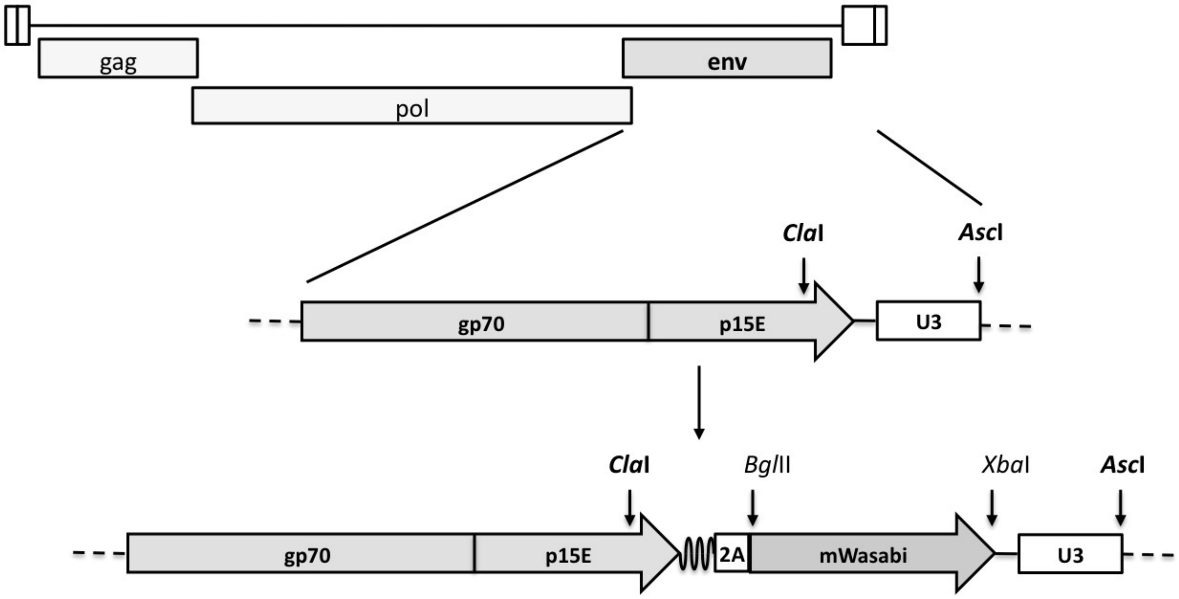

Supplementary Figure 1. Schematic representation of the mWasabi-encoding F-MuLV. The coding sequence of mWasabi, flanked by BglII and XbaI, was introduced at the C-terminus of the Env open reading frame, with sequences coding for a glycine-serine linker (indicated by zigzagging line) and the porcine teschovirus-derived self-cleaving 2A peptide between the Env p15E and the mWasabi sequences. ClaI and AscI sites were used for introduction of the modified sequence into the F-MuLV molecular clone pFB29.

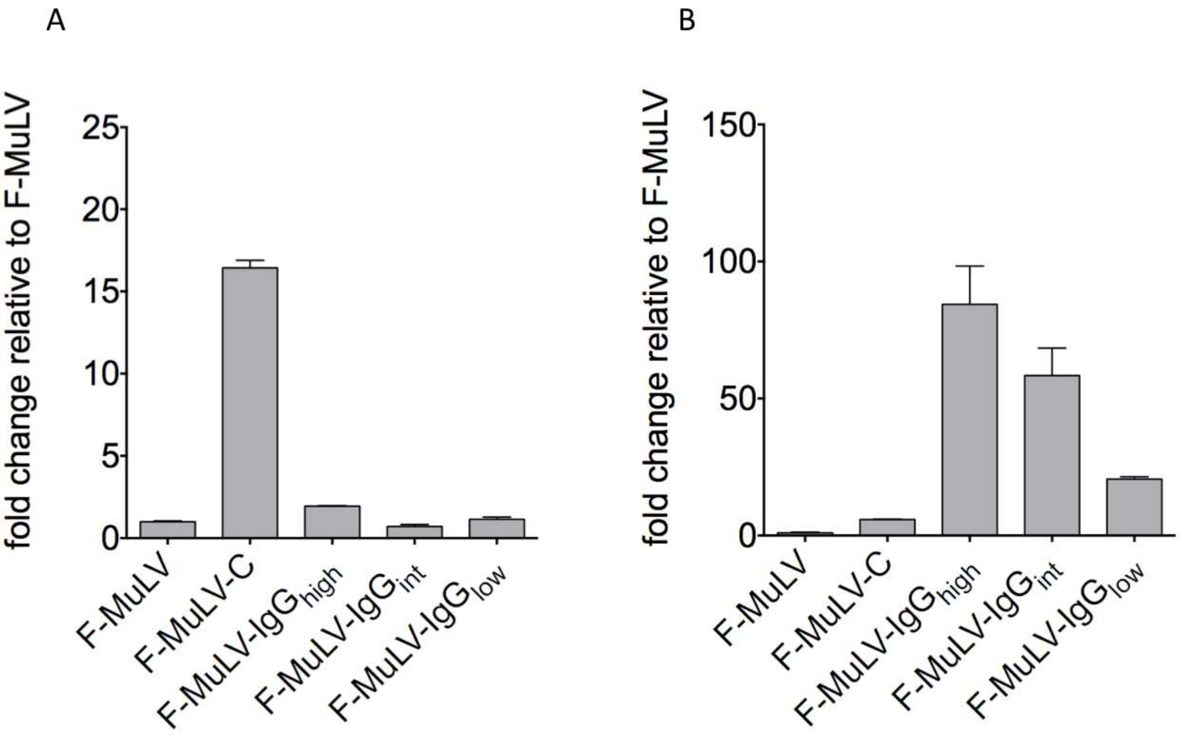

Supplementary Figure 2. VCA demonstrates the presence of (A) C3-fragments on complement-opsonized (F-MuLV-C) and (B) IgG molecules on Ab-opsonized (F-MuLV-IgG) virus. F-MuLV was opsonized either in the presence of normal mouse serum (NMS) as source of complement at a dilution

of 1:10 for 60 min at 37°C (F-MuLV-C), or an IgG opsonization of F-MuLV (F-MuLV-IgG) was done by incubation of the virus with 5 µg/ml (F-MuLV-IgG_{high}), 0.5 µg/ml (F-MuLV-IgG_{int}) or 0.05 µg/ml (F-MuLV-IgG_{low}) of F-MuLV envelope-specific non-neutralizing monoclonal antibody clone #48 [31] for 60 min at 37°C. As control, F-MuLV was in cubated in medium alone. After opsonization to remove NMS and unbound IgG virus was ultracentrifuged (23.000×g, 2 hrs, 4°C) and the virus pellet was resuspended in RPMI. Opsonization pattern was determined in a virus capture assay using a 96-well ELISA plate coated with rabbit anti-mouse IgG or rabbit anti-mouse C3 antibodies. Equal amount of viral RNA according to RT-PCR results from differentially opsonised virus stocks was added and the plate was incubated overnight at 4°C. Then unbound virus was removed by washing and RNA from bound virus was determined by RT-PCR. Data show mean±SEM of duplicate measurements.

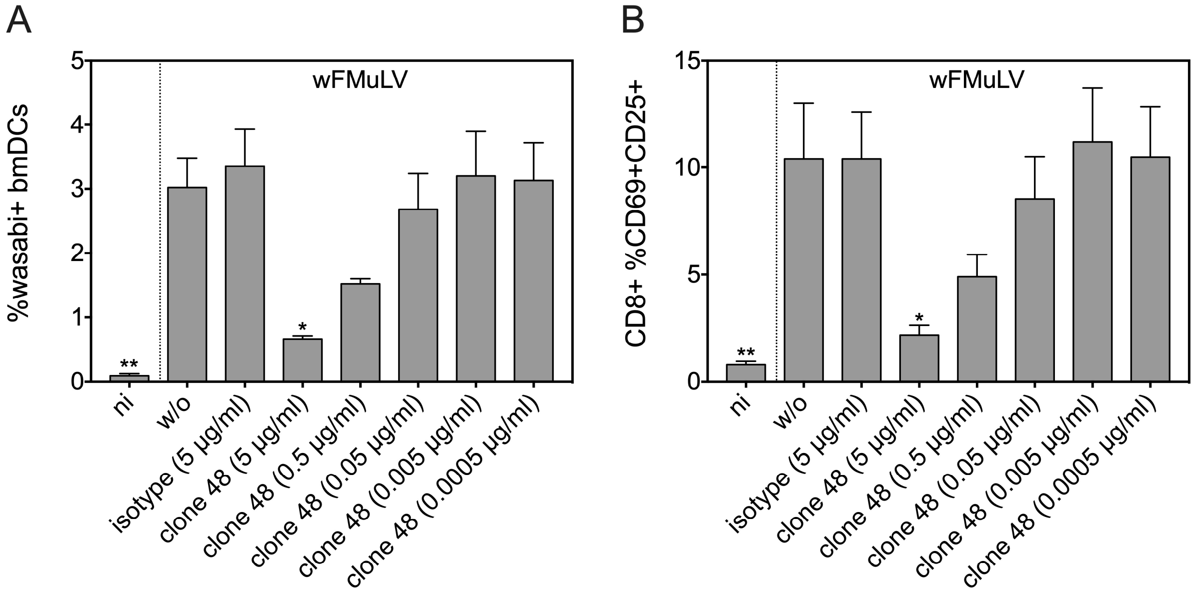

Supplementary Figure 3. FV-specific IgG-mediated, concentration dependent abrogation of bmDC infection by wF-MuLV correlates with decreased capacity of DCs to activate virus-specific CD8 T cells. (A) 5×10^5 DCs derived from bone marrow of C57BL/6 mice were infected with 5.000 FFU mWasabi-encoding F-MuLV (wF-MuLV) in the presence or absence of different concentrations of FV-specific clone 48 (from 5 to 0.0005 µg/ml) or isotype (5 µg/ml) antibodies. Cells were further cultivated for 2 days at 37°C and mWasabi positive infected cells were determined by FACS. **(B)** Infected DCs were subsequently cocultured with 5×10^5 FV-specific TCRtg CD8 T cells. Activation of CD8 T cells was determined by the expression of CD25 and the early activation marker CD69 gating on AAD negative living CD3+CD8+ singlets measured by flow cytometry after 48 hours of coculture. Mean±SEM derived from 3 independent experiments are shown. Data were analyzed by GraphPad PRISM software using ANOVA followed by Dunnet's multiple comparisons test (**,* significant to wF-MuLV w/o at $p<0.01$, $p<0.05$, respectively).

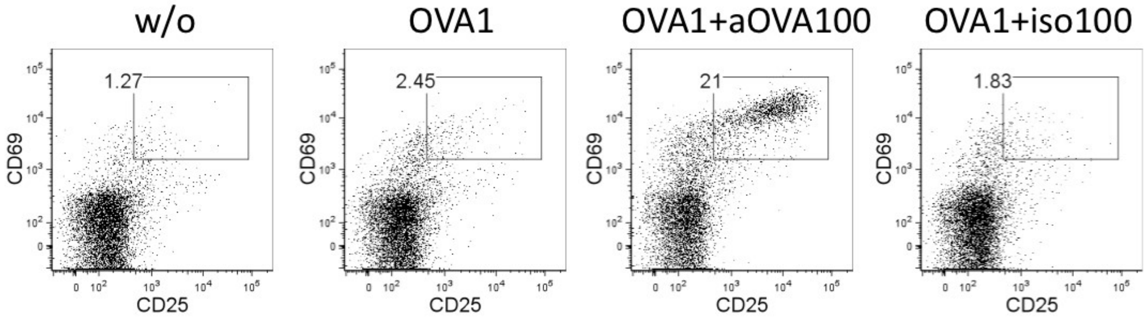

Supplementary Figure 4. Immune-complexed IgG-OVA facilitates DC-mediated activation of OVA-specific OT-1 CD8 T cells. Ovalbumin (1 µg/ml, OVA1) was preincubated for 10 min either alone or in the presence of ovalbumin-specific Abs (100 µg/ml, aOVA100) or isotype Abs (100µg/ml, iso100) to generate OVA-ICs. OVA or OVA-ICs were then loaded to 5×10^5 bmDCs and incubated overnight at 37°C. Then, bmDCs were washed and cocultured with 5×10^5 isolated OVA-specific CD8 TCR tg OT-1 T cells for 48 hrs. After 48 hours of co-cultivation activation of CD8 T cells was determined by the expression of CD25 and the early activation marker CD69 gating on AAD negative living CD8 singlets measured by FACS.
